# A loss of c-kit expression is associated with an advanced stage and poor prognosis in breast cancer

**DOI:** 10.1038/sj.bjc.6603183

**Published:** 2006-05-23

**Authors:** S Tsutsui, K Yasuda, K Suzuki, H Takeuchi, T Nishizaki, H Higashi, S Era

**Affiliations:** 1 Department of Breast Surgery, Matsuyama Red Cross Hospital, 1 Bunkyo, Matsuyama 790-8524, Japan; 2 Department of Surgery, Beppu Medical Center, Beppu, Japan; 3 Department of Surgery, Matsuyama Red Cross Hospital, Matsuyama, Japan; 4 Department of Pathology, Beppu Medical Center, Beppu, Japan

**Keywords:** breast cancer, c-kit, prognosis

## Abstract

To evaluate the c-kit expression in breast cancer, 217 invasive ductal carcinomas of the breast were immunohistochemically stained for c-kit protein. The c-kit expression was positive in 59 (27%) of 217 tumours, while the c-kit expression was negative in 158 (73%) of 217 tumours. There was a significant correlation between a negative expression of the c-kit protein and lymph node metastasis (*P*<0.0001), and the incidence of a negative expression of the c-kit protein increased as the number of the metastatic lymph nodes increased (*P*=0.0003). The c-kit expression did not significantly correlate with the tumour size, nuclear grade, oestrogen receptor status, MIB-1 counts and p53 protein expression. A univariate analysis indicated the patients with the negative c-kit expression to have a worse disease-free survival (DFS) than those with the positive c-kit expression (*P*=0.0041), while a multivariate analysis determined lymph node metastases and the MIB-1 counts to be independently significant factors for DFS. In conclusion, a loss of the c-kit expression was found in about three-fourth of invasive ductal carcinoma of the breast and was associated with lymph node metastases. The prognostic implications of the c-kit expression seem to be due to fact that a loss of the c-kit expression is associated with an advanced stage of breast cancer.

The c-kit proto-oncogene encodes a transmembrane tyrosine kinase receptor, sharing a structural similarity with the platelet-derived growth factor and colony-stimulating factor-1 receptor (Yarden *et al*, 1987; Qiu *et al*, 1988). The c-kit is activated by its ligand, stem cell factor, and plays various roles in haematopoiesis, melanogenesis, spermatogenesis and the development of the interstitial cells of Cajal (Ronnstrand, 2004). The c-kit is physiologically expressed in haematopoietic stem cells, tissue mast cells, germ cells, melanocytes, interstitial cells of Cajal and mammary gland epithelium, whereas the c-kit is not usually expressed in the normal squamous epithelium and the glandular epithelium of the lung, endocervix, pancreas, prostate, stomach and small and large intestines (Matsuda *et al*, 1993; Lammie *et al*, 1994). The diverse expressions of the c-kit in the normal tissues result in the diverse expressions of the c-kit in the tumour tissue originated from such normal tissues (Matsuda *et al*, 1993; Lammie *et al*, 1994; Gibson and Cooper, 2002). There have been several studies regarding the c-kit expression of breast cancer, indicating that the c-kit expression decreased in breast cancer tissue whereas the normal epithelium of the mammary gland showed the c-kit expression (Natali *et al*, 1992a; Matsuda *et al*, 1993; Hines *et al*, 1995; Chui *et al*, 1996; Palmu *et al*, 2002; Tsuura *et al*, 2002; Ko *et al*, 2003; Simon *et al*, 2004; Ulivi *et al*, 2004; Yared *et al*, 2004). There have, however, so far been few studies on the relationship between the c-kit expression of breast cancer and clinicolpathological factor such as lymph node metastasis and the proliferative activity, and the prognostic value of the c-kit expression in breast cancer has not yet been evaluated. Therefore, the aim of the present study was to evaluate the relationship between the c-kit expression and the clinicopathological factors in invasive ductal carcinoma of the breast, and the prognostic value of the c-kit expression in breast cancer was also evaluated using univariate and multivariate analyses.

## PATIENTS AND METHODS

### Patients

This study comprised 217 women with breast cancer who underwent surgery for breast cancer, without any evidence of distant metastasis at the time of surgery, between 1985 and 1998 at the Beppu Medical Center. The histological type of breast cancer in all patients was invasive ductal carcinoma, while any types other than invasive ductal carcinoma were excluded in this study. No cases of noninvasive carcinoma were included in this study. The patients' ages ranged from 23 to 86 years, with a mean age of 57.0 years. The patients were either treated by a mastectomy (186 patients) or by breast conservation treatment (31 patients). Lymph nodes dissection was performed in 216 patients. Adjuvant postoperative hormone therapy was given to 190 patients and 190 patients received adjuvant chemotherapy, while 46 patients received postoperative radiotherapy. The median follow-up duration was 6.59 years.

### Immunohistochemistry

**Figure 1:**
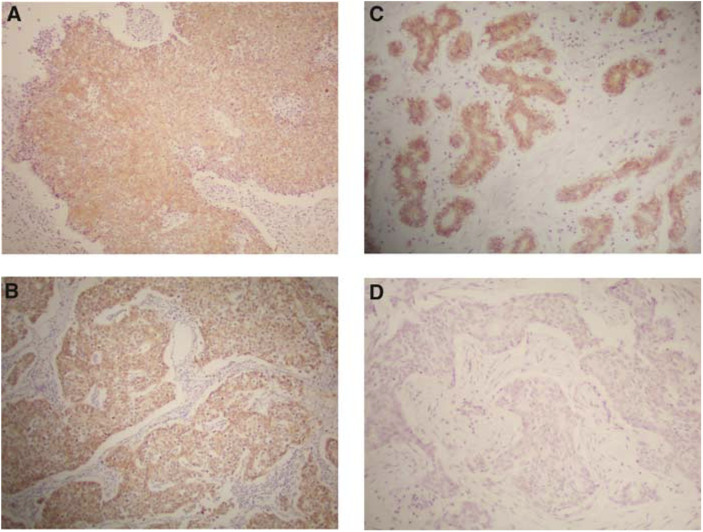
Immunohistochemical expression of c-kit protein in breast cancer. Two cases with a positive expression of the c-kit protein in breast cancer cells (**A** and **B**). A case with a negative expression of c-kit protein (**C**: normal mammary gland and **D**: breast cancer cells in the same slide), in which the expression of c-kit protein of breast cancer cells (**D**) was negative, in comparison to the expression of the c-kit protein in the normal mammary gland (**C**).

For an immunohistochemical analysis of the c-kit protein, 3-ìm sections were dewaxed and rehydrated, and antigen retrieval was performed by microwave heating for 15 min in a 10 mM citrate buffer at pH 6.0. Next, the sections were reacted with rabbit polyclonal antibody for c-kit (A4502, DAKO, Kyoto, Japan) diluted at 1 : 100 for 60 min at room temperature, and then were subsequently stained by the universal immuno-peroxidase polymer method using a Histofine Simple Stain MAX PO(M) kit (Nichirei Corp., Tokyo, Japan), according to the protocol provided by the manufacturer. Positive reactions were visualised with diaminobenzidine, followed by counterstaining with haematoxylin. A normal mammary gland was used as an internal control for c-kit protein expression in the present study, since the normal mammary gland was demonstrated to show the c-kit protein expression (Natali *et al*, 1992a; Tsuura *et al*, 2002; Ulivi *et al*, 2004; Yared *et al*, 2004). Any slides in which there was no normal mammary gland or slides in which a normal mammary gland did not express an adequate degree of immunohistochemical staining of the c-kit protein were excluded in the present study. The immunohistochemical expression of breast cancer cells was judged to demonstrate either a positive or negative expression, in comparison to the c-kit protein expression of the normal mammary gland (Figure 1). The c-kit protein expression was independently determined by three authors (ST, KY and SE) in whom two authors did not know any clinicopathological information for each patient.

We also examined any correlations between the c-kit expression and other biological parameters for cell cycle regulation (p53 protein) and cell proliferation (MIB-1 counts) that have been previously studied by immunohistochemistry for the tumours in the present series (Tsutsui *et al*, 2004). The immunohistochemical procedures and results for these parameters have all been described previously (Tsutsui *et al*, 2004).

### Statistical analysis

The *χ*^2^ test was used to investigate the significance of the relationships between the c-kit expression and individual variables. The disease-free survival (DFS) was estimated using the Kaplan and Meier method, and any differences in the survival curves were compared by the Log-rank test. A multivariate analysis was performed by Cox's proportional hazards model. The *P*-value of <0.05 was regarded as statistically significant. All statistical analyses were performed using the StatView 5.0 software package (SAS institute Inc. Cary, NC, USA).

## RESULTS

The immunohistochemical expression of the c-kit protein of breast cancer was determined to be positive in 59 (27%) of 217 tumours, while the c-kit expression was negative in 158 (73%) of 217 tumours. The relationships between the c-kit expression and clinicopathological factors in the 217 breast cancers are shown in Table 1. There was a significant correlation between a negative expression of the c-kit protein and lymph node metastasis (*P*<0.0001), while the c-kit expression did not significantly correlate with tumour size, nuclear grade, oestrogen receptor status, MIB-1 counts, and p53 protein expression. Table 2 shows the relationship between the c-kit expression and the number of the metastatic lymph nodes. The incidence of a negative expression of the c-kit protein increased as the number of the metastatic lymph nodes increased (*P*=0.0003).

**Figure 2:**
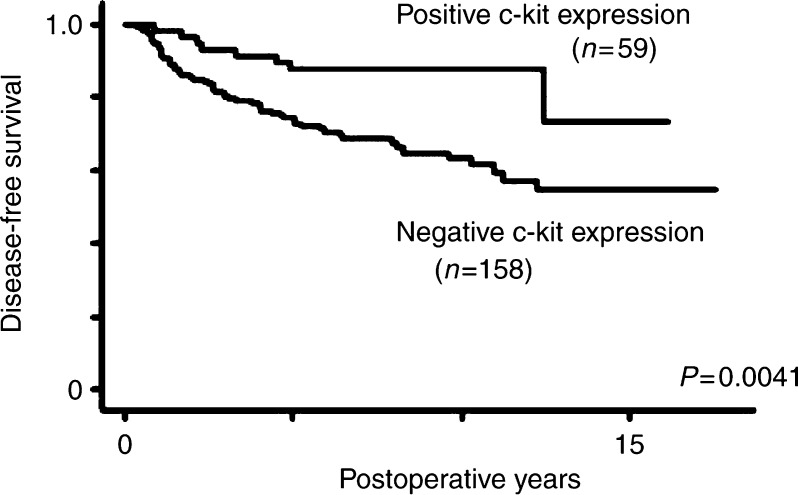
Disease free survival curve stratified according to the c-kit expression.

A univariate analysis indicated the 158 patients with a negative c-kit expression to have a significantly (*P*=0.0041) worse DFS than the 59 patients with a positive c-kit expression (Figure 2). In the 117 patients negative for lymph node metastasis, no significant difference was observed in the DFS of the patients between a positive and negative c-kit expression. A multivariate analysis determined lymph node metastasis and the MIB-1 counts to be independently significant factors for DFS, while the c-kit expression was not an independently significant (*P*=0.3108) factor for DFS (Table 3).

## DISCUSSION

Gastrointestinal stromal tumour (GIST) was demonstrated to be frequently associated with mutations in the c-kit gene and an activating mutation in exon 11 of the c-kit gene is considered to be a causative factor for GIST (Hirota *et al*, 1998). On the other hand, no activating mutation of c-kit was found in breast cancer (Simon *et al*, 2004) and c-kit is physiologically expressed in the normal gland of the breast (Natali *et al*, 1992a; Tsuura *et al*, 2002; Ulivi *et al*, 2004; Yared *et al*, 2004). In the present study, a normal mammary gland in the same slide was therefore used as an internal control for the immunohistochemical expression of c-kit protein, while a loss of the c-kit protein expression of the cancer cells was determined in comparison with the c-kit protein expression of normal mammary glands. Similar findings, indicating that c-kit is involved in the growth and maintenance of the normal epithelium and the c-kit function may be lost following malignant transformation (Natali *et al*, 1992a; Matsuda *et al*, 1993), have also been demonstrated in melanoma (Natali *et al*, 1992b) and thyroid (Natali *et al*, 1995) and renal (Miliaras *et al*, 2004) cancers.

The published series of studies regarding c-kit expression in the breast are summarised in Table 4. The rate of a positive c-kit expression in breast cancer varies from 1 to 82%, which is likely attributable to the different methods for determining the c-kit expression. The rate of a positive c-kit expression in the normal breast tissue was all 100% in four studies (Natali *et al*, 1992a; Tsuura *et al*, 2002; Ulivi *et al*, 2004; Yared *et al*, 2004). Although the rate of a positive c-kit expression varied in the different studies, the rate of a positive c-kit expression of benign tumours was always lower than that of normal breast tissue and the rate of a positive c-kit expression of the breast cancer was always lower than that of benign tumours in each study (Natali *et al*, 1992a; Tsuura *et al*, 2002; Yared *et al*, 2004). In two studies in which the scoring method was used for the immunohistochemical staining (Chui *et al*, 1996; Ko *et al*, 2003), the immunoreactive score (IRS) of the c-kit expression of benign tumours was lower than that of normal breast tissue and the IRS of the breast cancer was significantly lower than that of the benign tumours. The finding of the c-kit expression of breast cancer is quite a contrast to the finding of EGFR and erbB2 expression of breast cancer, in which the overexpression of EGFR and erbB2 indicated a malignant phenotype of breast cancer (Tsutsui *et al*, 2002a, 2002b).

The survival analyses for the c-kit expression have been demonstrated in several malignant tumours (Tonary *et al*, 2000; Bar-Sela *et al*, 2003; Scobie *et al*, 2003; Krams *et al*, 2004; Simon *et al*, 2004; Pan *et al*, 2005), while there was only one survival analysis (Simon *et al*, 2004) regarding breast cancer, in which no difference was found in the survival between the patients with or without the c-kit expression. Although no statistically significant difference in clinical outcomes has been found in endometrial adenocarcinomas (Scobie *et al*, 2003), nasopharygeal carcinomas (Bar-Sela *et al*, 2003) and small cell carcinomas of the urinary bladder (Pan *et al*, 2005), two studies on ovarian cancers (Tonary *et al*, 2000) and neuroblastomas (Krams *et al*, 2004) indicated an association between a loss of the c-kit expression and poor prognosis. In ovarian cancers, the loss of c-kit expression was associated with a poor prognosis, while the c-kit expression tended to decrease at an advanced stage (Tonary *et al*, 2000), being similar with our findings in breast cancers. On the other hand, the prognostic value of the c-kit expression was demonstrated based on multivariate analyses for nueroblastomas (Krams *et al*, 2004). In the present study, a univariate analysis demonstrated the prognosis of the patients with a loss of the c-kit expression to be significantly worse than those with a positive c-kit expression, while a multivariate analysis demonstrated lymph node metastasis and a proliferative activity determined by MIB-1 counts, but not the c-kit expression, to be independently significant prognostic factors for DFS. There was a close correlation between a loss of the c-kit expression and lymph node metastasis in the present study, while no significant correlation between the c-kit expression and lymph node metastasis was found in other studies (Chui *et al*, 1996; Simon *et al*, 2004; Ulivi *et al*, 2004). In the present study, the c-kit expression did not correlate with the MIB-1 counts. The findings in the present study indicated that a loss of the c-kit expression was associated with an advanced stage of breast cancer, but not with a proliferative activity of the cancer cells, and also suggested that a close correlation between a loss of the c-kit expression and an advanced stage of breast cancer seems to mainly account for the prognostic implications of the c-kit expression in breast cancer.

In conclusion, we demonstrated a loss of c-kit expression, as demonstrated by immunohistochemistry, to occur in about three-fourths of all breast cancers. A loss of the c-kit expression correlated with lymph node metastasis and the incidence in the loss of the c-kit expression increased as a number of metastatic lymph nodes increased. The prognostic value of the c-kit expression was demonstrated by a univariate analysis, but not by a multivariate analysis. The prognostic implications of the c-kit expression therefore seem to be due that a loss of the c-kit expression is associated with an advanced stage of breast cancer.

## Figures and Tables

**Table 1:** The c-kit expression and clinicopathological factors in breast cancer

		**c-kit expression**				
		**Negative**		**Positive**		
	**No. of patients**	***n*=158**	**(%)**	***n*=59**	**(%)**	***P*-value**
*Age*						0.3807
<50	71	49	(69)	22	(31)	
50⩽	146	109	(75)	37	(25)	
						
*Tumour size (cm)*						0.1532
–2.0	54	34	(63)	20	(37)	
2.1–5.0	138	104	(75)	34	(25)	
5.1–	25	20	(80)	5	(20)	
						
*Lymph node metastasis* a					<0.0001	
Absent	117	72	(62)	45	(38)	
Present	99	85	(86)	14	(14)	
						
*Nuclear grade*						0.1188
I or II	148	103	(70)	45	(30)	
III	69	55	(80)	14	(20)	
						
*Oestrogen receptor*						0.6139
Positive	86	61	(71)	25	(29)	
Negative	131	97	(74)	34	(26)	
						
*MIB-1 counts*						0.4333
Negative (<10%)	127	95	(75)	32	(25)	
Positive (10%⩽)	90	63	(70)	27	(30)	
						
*P53 protein expression*					0.6025	
Negative	160	118	(74)	42	(26)	
Positive	57	40	(70)	17	(30)	

a Lymph node dissection was performed in 216 patients.

**Table 2:** The c-kit expression and the number of the metastatic lymph nodes in breast cancer

		**c-kit expression**				
		**Negative**		**Positive**		
**No. of the metastatic lymph nodes**	**No. of patients**	***n*=157**	**(%)**	***n*=59**	**(%)**	***P*-value**
						0.0003
0	117	72	(62)	45	(38)	
1–3	60	48	(80)	12	(20)	
4–9	16	15	(94)	1	(6)	
10–	23	22	(96)	1	(4)	

**Table 3:** Multivariate analyses for DFS

**Variables**	***P*-value**	**Relative risk**	**95% CI**
*Age*			
50⩽ (*vs* <50)	0.4498	0.82	0.48–1.38
			
*Tumour size (cm)*			
2.1–5.0 (*vs* −2.0)	0.8303	0.92	0.42–2.01
5.1–(*vs* −2.0)	0.1458	1.99	0.79–5.05
			
*Lymph node metastasis*			
Present (*vs* absent)	<0.0001	5.94	2.95–12.0
			
*Nuclear grade*			
III (*vs* I or II)	0.0732	1.65	0.95–2.85
			
*Oestrogen receptor*			
Negative (*vs* positive)	0.6538	1.14	0.65–1.99
			
*MIB-1 counts*			
10%⩽ (*vs* <10%)	0.0142	2.02	1.15–3.53
			
*P53 protein expression*			
Positive (*vs* negative)	0.2028	1.50	0.80–2.79
			
*c-kit expression*			
Negative (*vs* positive)	0.3108	1.51	0.68–3.33

**Table 4:** Publised series regarding the c-kit expression for breast tissue

		**c-kit expression**				
**Author (year)**	**Method**	**Normal breast tissue**	**Benign tumours**	***In situ* cancers**	**Invasive primary cancers**	**Metastatic breast cancers**
Natali *et al* (1992a, 1992b)	IHC^a^	6/6 (100%)	28/36 (78%)		10/80 (13%)	1/40 (3%)
						
Matsuda *et al* (1993)	IHC				2/10 (20%)	
						
Hines *et al* (1995)	mRNA IHC				9/11 (82%)	
						
Chui *et al* (1996)	IHC (IRS^b^)	6.22+2.11 (*n*=20)	3.33+2.44 (*n*=58)		0.43+1.27 (*n*=57)	
						
Tsuura *et al* (2002)	IHC	338/338 (100%)	131/141 (93%)	0/11 (0%)	2/171 (1%)	
						
Palmu *et al* (2002)	IHC				33/40^c^ (82%)	
						
Ko *et al* (2003)	IHC (IRS)	5.90+1.37 (*n*=20)	4.05+1.82 (*n*=20)		0.90+1.79 (*n*=40)	1.06+1.86^d^ (*n*=18)
						0.20+0.63^e^ (*n*=10)
						
Yared *et al* (2004)	IHC	21/21 (100%)	16/24 (88%)	3/29 (10%)	4/41 (10%)	0/4 (0%)
						
Ulivi *et al* (2004)	IHC	14/14 (100%)		7/16 (44%)	7/75 (9%)	
						
	mRNA	14/14 (100%)		12/16 (75%)	3/14 (21%)	
						
Simon *et al* (2004)	IHC				43/1654 (2.6%)	

a Imuunohistochemistry.

b Immunoreactive score.

c All patients have progressive metastatic breast cancer.

d Average in the metastatic lymph nodes.

e Average in distant metastases.
